# Best regimens for treating chemo‐naïve incurable squamous non‐small cell lung cancer with a programmed death‐ligand 1 tumor proportion score of 1%–49%: A network meta‐analysis

**DOI:** 10.1111/1759-7714.14229

**Published:** 2021-11-17

**Authors:** Nobuhiko Fukuda, Nobuyuki Horita, Ho Namkoong, Ayami Kaneko, Kohei Somekawa, Yoichi Tagami, Keisuke Watanabe, Yu Hara, Nobuaki Kobayashi, Takeshi Kaneko

**Affiliations:** ^1^ Department of Pulmonology Yokohama City University Graduate School of Medicine Yokohama Japan; ^2^ Department of Infectious Diseases Keio University School of Medicine Tokyo Japan

**Keywords:** immune checkpoint inhibitors, lung neoplasms, molecular targeted therapy, systematic review

## Abstract

**Background:**

Non‐small cell lung cancer (NSCLC) is the leading cause of cancer‐related mortality worldwide. It is advisable to select the appropriate treatment based on characteristics of the cancer such as pathology, mutations, and programmed death‐ligand 1 (PD‐L1) levels. In this study, by remarking squamous NSCLC with low PD‐L1 expression without mutations, we investigated the efficacy and safety of regimens that included molecularly targeted drugs such as immune checkpoint inhibitors (ICIs) through a network meta‐analysis.

**Methods:**

Databases were searched systematically to identify appropriate articles, in which randomized trials with incurable squamous NSCLC were described. Suitable studies were manually checked by two reviewers. A random model network meta‐analysis was conducted, in which the primary outcome was the overall survival rate.

**Results:**

We identified 48 studies, which included 16 391 patients. When a platinum + third‐generation cytotoxic agent regimen (platinum regimen) was a reference, the platinum regimen + pembrolizumab (Pemb) yielded the best results in regard to the overall survival rate when compared with chemotherapy (hazard ratio [HR] = 0.57, 95% confidence interval [CI] = 0.36–0.90, *p* = 0.016) followed by the platinum regimen + nivolumab (Niv) + ipilimumab (Ipi) (HR = 0.61, 95% CI = 0.44–0.84, *p* = 0.003). However, the efficacy of ICI monotherapy was not statistically different from that of the platinum regimen.

**Conclusions:**

The combination therapies, which were the platinum regimen + Pemb and the platinum regimen + Niv + Ipi, rather than ICI monotherapy were effective first‐line agents for treating squamous NSCLC with low PD‐L1 levels.

## Graphical abstract

Clinicians wonder which treatment is best for patients with squamous lung cancer expressing low programmed death‐ligand 1 and no mutations. This study presents the comparison of regimens including immune checkpoint inhibitors using network meta‐analysis with 48 studies and 16 391 patients. The primary outcome was overall survival. Our main result was that the platinum regimen + pembrolizumab was the best regimen (hazard ratio = 0.57, 95% confidence interval = 0.36–0.90, *p* = 0.016).

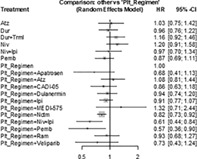

## INTRODUCTION

Non‐small cell lung cancer (NSCLC) is the leading cause of mortality in the world.^1^ Platinum doublet chemotherapy is the standard of care for patients diagnosed with inoperable NSCLC lacking sensitizing mutational drivers. Immune checkpoint inhibitors (ICIs) have broadened the options of cancer treatment with a new mechanism that suppresses immune evasion by tumor co‐suppressors and activates antitumor immunity.^2^ For example, programmed cell death protein and cytotoxic T‐lymphocyte‐associated antigen 4 are immune checkpoint proteins in lung cancer.^3^ According to recent guidelines, drugs that are used for combination therapy or monotherapy are considered for each programmed death‐ligand 1 (PD‐L1) expression and histological type.^4^, ^5^, ^6^, ^7^ The expression of PD‐L1 is classified as a tumor proportion score (TPS) of 50% or higher, 1%–49%, and <1%. NSCLCs are classified predominantly as adenocarcinoma and squamous cell carcinoma. These two types differ in smoking history, tumor location, and clinical outcomes.^8^ Appropriate treatment must be selected based on the characteristics of cancer. Incidentally, kinase inhibitors such as target epidermal growth factor receptor (EGFR), anaplastic lymphoma kinase, B‐Raf proto‐oncogene, and ROS proto‐oncogene 1 are specifically effective in NSCLC with mutations, but these are not recommended for patients who do not have these mutations.^9^

The superiority of regimens is often determined in randomized clinical trials (RCTs), however, all regimens, especially ICI regimens, are not compared. Understanding the differences between ICI regimens is important during the process of choosing a treatment. Network meta‐analysis is an optimal analytical method that allows for indirect comparisons among multiple regimens. In 2017, we conducted a network meta‐analysis of cytotoxic regimens for first‐line incurable NSCLC.^10^ At this time, remarking squamous NSCLC with low PD‐L1 expression (TPS 1%–49%) and no mutations, we investigated the efficacy and safety of regimens including molecularly targeted drugs, such as ICIs, by conducting a network meta‐analysis.

## METHODS

### Protocol registration

The protocol of our analysis conformed to the Preferred Reporting Items for Systematic Reviews reporting checklist^11^ and was registered at the University Hospital Medical Information Network Center, Japan.^12^ The contents of the protocol have been partially revised (Text S1). In the systematic review, informed consent of the patient and approval of the institutional review board was not required.

### Study search

To identify eligible articles, the MEDLINE, Web of Science Core Collection, Embase, and Cochrane Central Register of Controlled Trials databases were searched systematically on October 15, 2020. The equations used in MEDLINE are listed in Text S2. The titles and abstracts of the shortlisted papers were screened, and those that met the inclusion criteria were reviewed in the full text. The suitable studies were manually checked by two review authors (N.F. and K.S.). Any dispute in the selection process was discussed and resolved by the two reviewers (NF and N.H.).

### Selection criteria: publication type and trial design

The published sources had to be written in English. RCTs that enrolled patients with advanced NSCLC were included in this study. Trials that focused on NSCLC with oncogenic mutations were excluded. Conference abstracts were allowed. Studies that included treatment only in the form of first‐line chemotherapy were included. Studies that included maintenance therapy, second‐line therapy, and later‐line therapy were excluded.

### Selection criteria: chemotherapy

Appropriate treatments included platinum doublet chemotherapy, ICIs, and molecularly targeted therapies. Clinical studies on platinum plus an angiogenesis inhibitor have also been conducted. ICI can be administered alone or in combination with platinum‐based treatments.

There is a controversy regarding the difference between cisplatin (CDDP) and carboplatin (CBDCA),^13^, ^14^ but in recent clinical trials, physicians can choose either CDDP or CBDCA.^15^, ^16^ Keynote 407, which is one of the material RCTs related to pembrolizumab (Pemb),^17^ paclitaxel (Ptx), and nanoparticle albumin‐bound Ptx (nabPtx), was considered similarly. For this reason, CDDP/CBDCA and Ptx/nabPtx were considered identical during analysis. Nedaplatin expression was also differentiated.

A network meta‐analysis based on individual regimens cannot evaluate the physicians' choice regimens. Two models were established to assay these items. The “main model” did not make a distinction between CDDP or CBDCA plus third‐generation cytotoxic drug regimen (platinum regimen). The “separate model” evaluated each platinum regimen differently.

Because the use of kinase inhibitors in patients without driver‐gene mutations is not standardized, they were excluded from the study.^4^, ^5^, ^6^, ^7^ Trials that examined pre‐ or post‐operative chemotherapy and concomitant chemoradiation were also excluded from this analysis.

### Selection criteria: patients

RCTs enrolled patients with advanced or recurrent squamous NSCLC. Regardless of whether classification of the tumor‐lesion‐metastasis was revised in the past, we adopted the stage described in the article. Patients with a history of surgery or radiotherapy were included in the study. Studies that focused on those with a low performance status or the elderly were excluded.

NSCLCs with a low or high PD‐L1 level (<1% or >50%) were excluded because recent guidelines have used the cut‐off of PD‐L1 levels at 1% or 50%.^4^, ^5^, ^6^, ^7^ If a portion of the surveyed population met our criteria, we analyzed that data subset. If a study provided data for three separate populations with TPSs of 0%, 1%–49%, and ≥50%, the data for those with a TPS of 1%–49% were used.

Trials focusing on non‐squamous carcinoma, oncogenic mutations, and patients with a TPS ≥50% were excluded. However, we accepted studies that did not clearly describe the pathological classification, oncogenic mutations, and PD‐L1 expression. This was necessary, because the majority of studies related to NSCLC would have been excluded otherwise. It has been reported that there are differences in PD‐L1 assay positivity.^18^ We thought that these differences were slight and they could be taken into consideration.

### Quality assessment

The quality of each incorporated study was checked using the Cochrane risk of bias tool. This judgment tool evaluated selection, performance, detection, attrition, reporting, and other biases.^19^

### Outcomes

The primary outcome was overall survival (OS); the secondary endpoints were progression‐free survival (PFS), adverse events based on the Common Terminology Criteria for Adverse Events grade III or higher,^20^ and treatment‐related death. Hazard ratios (HRs) were used to evaluate the OS and the PFS. Odds ratios (ORs) were used to evaluate adverse events (ORae) (>grade III) and treatment‐related death. Disease progression was determined according to the Response Evaluation Criteria in Solid Tumors guidelines issued in 2000, which were revised in 2009.^21^ If possible, imaging evaluations performed by an independent reviewer were preferred.

### Data extraction

The data of studies including the first author name, publication year, phase, primary outcome, pathology, stage, performance status, regimens, sample size (if possible, intention‐to‐treat analysis), and trial outcomes were independently extracted by two reviewers (N.F. andK.S.). Discrepancies were resolved through mediation by a third reviewer (N.H.). Parmar's method was used to analyze survival data.^22^ The regimens were defined according to the drug compounding independently of the dose and schedule. The subset data were collected by subtraction using a fixed‐model meta‐analysis. The results of the “PD‐L1 1% to 49%” subgroup were calculated by subtracting data of the “PD‐L1 <1%” subgroup from data of the “PD‐L1 <50%” subgroup. Data on adverse events and treatment‐related deaths could be obtained from NSCLC with pathology or any PD‐L1 TPS because stratified data were seldom noted.

### Statistical analyses

Our study applied the frequentist weighted least‐squares approach random‐model network meta‐analysis.^23^ The OR and HR were calculated using log‐conversion. Platinum + Ptx was used as a common reference in the separate model because this regimen was recently evaluated in trials for the treatment of squamous carcinoma.^17^ The results were performed using R software (Command: netmeta, Package: netmeta).^24^ A *p* value of <0.05 was considered statistically significant.

## RESULTS

### Study search

**FIGURE 1:**
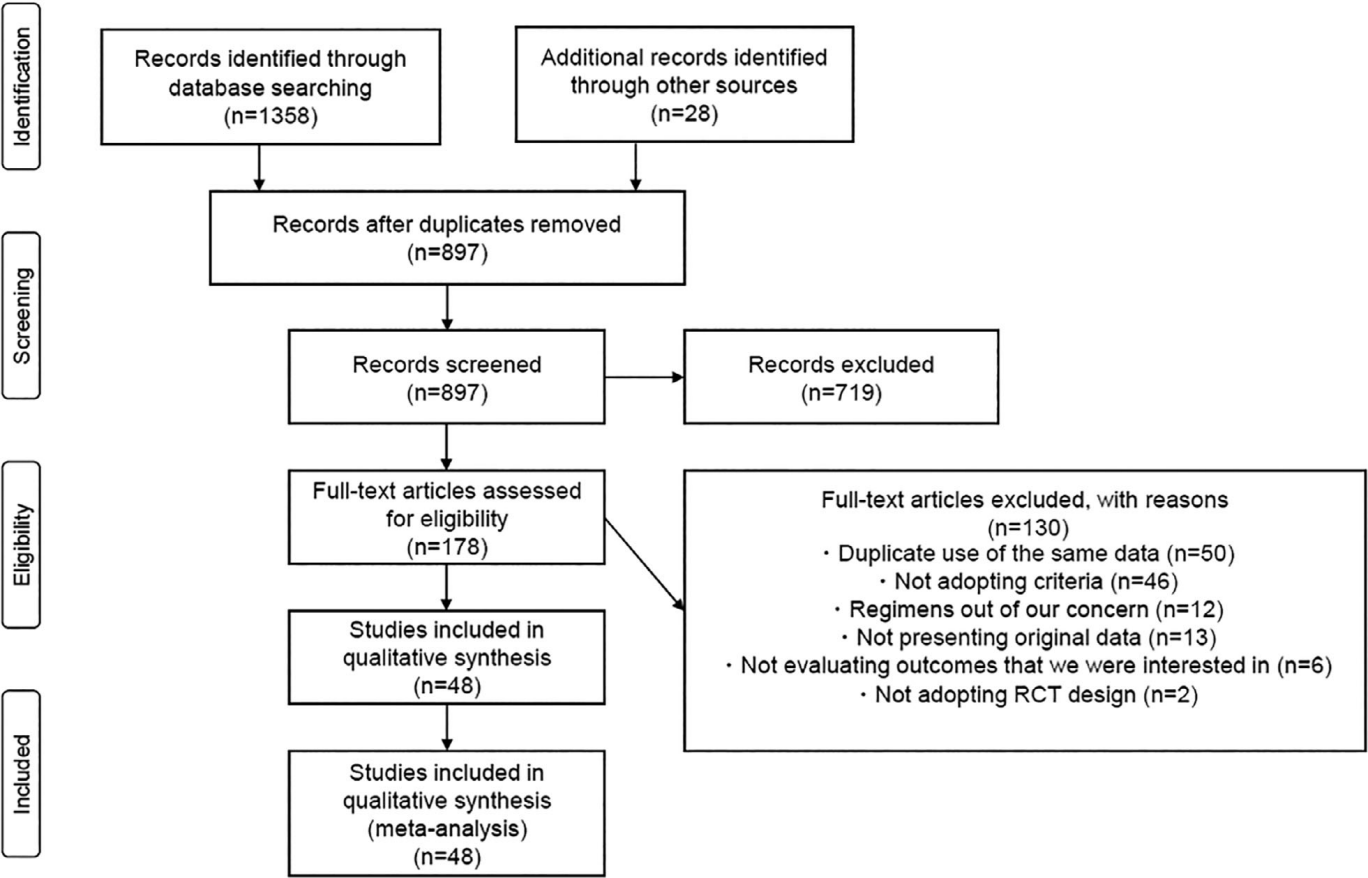
Preferred reporting items for systematic reviews and meta‐analyses flow chart for study search. RCT, randomized clinical trials

There were 1386 articles retrieved by electronic and hand searches. There were 897 articles that met the inclusion criteria. There were 489 articles removed because they were duplicate studies; 719 articles were removed during the process of screening title/abstract, and 130 articles were removed after reviewing the full article. Finally, 48 appropriate studies were identified (Figure 1; Supplementary References).

### Characteristics of the included studies

There were 19 studies and 39 arms, of which 11 included ICIs, in the main model and 42 studies and 89 arms, of which four included ICIs, in the separate model. The average age of the patients ranged from 51 to 67 years. A total of 16 391 patients were enrolled; the number of participants in the respective studies ranged from 50 to 1218 with a median of 261 participants (Table 1, Text S3). The Cochrane risk of bias evaluation indicated that all studies had at least one domain with a high risk of bias (Table S1).

**TABLE 1:** Characteristics of included studies

Study	Country	Phase	Primary outcome	Pathology	Arm	Stage	PS	Regimens	Pts	Median age
Belani 2017	India	NS	OS	NSCLC	2	IIIb, IV	ECOG 0–1	Cddp (100 mg/m^2^) + Ptx (175 mg/m^2^) + CADI‐05 (0.2 mL) Cddp (100 mg/m^2^) + Ptx (175 mg/m^2^) [M][S]	221	58
Carbone 2017	USA	III	PFS	NSCLC	2	IV, Rec	ECOG 0–1	Niv (3 mg/kg, q2w) 1 of 5 platinum doublets [M]	327	64
Chang 2008	China	NS	RR	NSCLC	2	IIIb, IV	ECOG 0–2	Cddp (80 mg/m^2^) + Gem (1000 mg/m^2^ [d 1,8,15]) Cddp (80 mg/m^2^) + Vnr (20 mg/m^2^ [d 1,8,15]) [S]	83	62
Chen 2007	Taiwan	II	RR	NSCLC	2	IIIb, IV	ECOG 0–2	Cddp (60 mg/m^2^) + Vnr (25 mg/m^2^ [d 1,8]) Cddp (60 mg/m^2^) + Dtx (60 mg/m^2^) [S]	94	63
Chen 2004	Taiwan	II	NS	NSCLC	2	IIIb, IV	ECOG 0–2	Cddp (60 mg/m^2^ [d15]) + Ptx (66 mg/m^2^ [d 1,8,15]) Cddp (60 mg/m^2^ [d15]) + Vnr (23 mg/m^2^ [d 1,8,15]) [S]	140	65
Comella 2000	Italy	III	OS	NSCLC	2†	IIIb, IV	ECOG 0–1	Cddp (120 mg/m^2^) + Vnr (30 mg/m^2^ [weekly]) Cddp (100 mg/m^2^) + Gem (1000 mg/m^2^ [d 1,8,15]) [S]	120	62
Douillard 2005	France	II	RR	NSCLC	2	IV	ECOG 0–2	Cddp (100 mg/m^2^) + Dtx (75 mg/m^2^) Cddp (100 mg/m^2^) + Vnr (30 mg/m^2^ [d 1,8]) [S]	239	57
Edelman 2004	USA	II	OS	NSCLC	2	IIIb, IV	ECOG 0–1	Cbdca (AUC 5.5) + Gem (1000 mg/m^2^[d 1,8]) Cddp (100 mg/m^2^) + Vnr (25 mg/m^2^ [d 1,8]) [S]	204	60
Fossella 2003	USA	III	OS (non‐inf)	NSCLC	3	IIIb, IV, Rec	KPS ≥70%	(Cddp [75 mg/m^2^] or Cbdca [AUC 6]) + Dtx (75 mg/m^2^) Cddp (100 mg/m^2^) + Vnr (25 mg/m^2^ [weekly]) [S]	1218	60
Gebbia 2010	Italy	II	AE	NSCLC	2	IIIb, IV	ECOG 0–1	Cddp (75 mg/m^2^) + Dtx (75 mg/m^2^) Cddp (80 mg/m^2^) + Vnr (30 mg/m^2^ [d 1,8]) [S]	86	62
Gebbia 2003	Italy	III	TTP & OS	NSCLC	2‡	IIIb, IV	ECOG 0–2	Cddp (100 mg/m^2^) + Vnr (25 mg/m^2^ [d 1,8]) Cddp (100 mg/m^2^) + Gem (1400 mg/m^2^ [d 1,8]) [S]	278	62
Govindan 2017	USA	III	OS	Sq	2	IV, Rec	ECOG 0–1	Cbdca (AUC 6) + Ptx (175 mg/m^2^) + Ipi (10 mg/kg) Cbdca (AUC 6) + Ptx (175 mg/m^2^) [M][S]	956	64
Grossi_2018	Italy	II	DCR	Sq	2	IIIb, IV, Rec	Not available	Cddp (80 mg/m^2^) + Vnr (Oral 60–80 mg d1,8) Cddp (75 mg/m^2^) + Gem (1250 mg/m^2^) [S]	113	63
Harada 2019	Japan	II	RR	Sq	2	IIIb, IV, Rec	Not available	Cbdca (AUC 6) + Ptx (75 mg/m^2^) Cddp (80 mg/m^2^) + Gem (1000 mg/m^2^) [S]	71	Not available
Helbekkmo 2007	Norwegian	III	OS	NSCLC	2	IIIb, IV	ECOG 0–2	Cbdca (AUC 5) + Vnr (25 mg/m^2^ [d 1,8]) Cbdca (AUC 5) + Gem (1000 mg/m^2^ [d 1,8]) [S]	444	67
Hellmann 2019	USA	III	OS	NSCLC	2†	IV, Rec	ECOG 0–1	Niv (240 mg/kg q2w or 360 mg/kg q3w) + Ipi (1 mg/kg q6w) (Cddp [75 mg/m^2^] or Cbdca [AUC5)] + Gem (1000 mg/m^2^)/Pemt (500 mg/m^2^) [M]	396	63
Herbst 2020	USA	III	OS	NSCLC	2	IV	ECOG 0–1	Atz (1200 mg) (Cddp [75 mg/m^2^] or Cbdca [AUC6]) + (Gem 1000–1250 mg/m^2^ for Sq or Pemt 500 mg/m^2^ for NSq) [M]	572	65
Jotte 2020	USA	III	PFS, OS	Sq	2†	IV	ECOG 0–1	Cbdca (AUC 6) + nabPtx (100 mg/m^2^ qw) + Atz (1200 mg) Cbdca (AUC 6) + nabPtx (100 mg/m^2^ qw) [M][S]	261	65
Kawahara 2013	Japan	II	PFS	NSCLC	2	IIIb, IV, Rec	ECOG 0–1	Cbdca (AUC 6) + Dtx (60 mg/m^2^) Cbdca (AUC 6) + Ptx (200 mg/m^2^) [S]	90	67
Khodadad 2014	Iran	NS	PFS	NSCLC	2	IIIb, IV	ECOG 0–2	Cddp (75 mg/m^2^) + Dtx (75 mg/m^2^) Cbdca (AUC 5) + Ptx (200 mg/m^2^) [S]	100	51
Kubota 2015	Japan	III	OS (non‐inf)	NSCLC	2	IIIb, IV, Rec	ECOG 0–1	Cddp (60 mg/m^2^ [d8]) + S1 (80 mg/m^2^ [d 1–14 po bid]) Cddp (80 mg/m^2^) + Dtx (60 mg/m^2^) [S]	608	62
Lu 2018	China	III	PFS	Sq	2	IIIb, IV, Rec	ECOG 0–1	Cdgp (80 mg/m^2^) + Dtx (75 mg/m^2^) Cddp (75 mg/m^2^) + Dtx (75 mg/m^2^) [S]	286	62
Martoni 2005	Italy	III	TTP	NSCLC	2	IIIb, IV, Rec	KPS ≥70%	Cddp (75 mg/m^2^) + Vnr (25 mg/m^2^ [d 1,8]) Cddp (75 mg/m^2^) + Gem (1200 mg/m^2^ [d 1,8]) [S]	286	63
Minami 2013	Japan	II	PFS	NSCLC	2	IIIb, IV	ECOG 0–1	Cbdca (AUC 6) + Ptx (200 mg/m^2^) Cbdca (AUC 5) + Gem (1000 mg/m^2^ [d 1,8]) [S]	50	64
Mok 2019_sq	Hong Kong	III	OS	Sq	2	IIIb, IV	ECOG 0–1	Pemb (200 mg) Cbdca (AUC 5–6) + Ptx (200 mg/m^2^) [M][S]	271	63
Ohe 2007	Japan	III	OS (non‐inf)	NSCLC	4	IIIb, IV	ECOG 0–1	Cddp (80 mg/m^2^) + Cpt‐11 (60 mg/m^2^ [d 1,8,15]) Cbdca (AUC 6) + Ptx (200 mg/m^2^) Cddp (80 mg/m^2^) + Gem (1000 mg/m^2^ [d 1,8]) Cddp (80 mg/m^2^) + Vnr (25 mg/m^2^ [d 1,8]) [S]	602	62
Okamoto 2010	Japan	III	OS (non‐inf)	NSCLC	2	IIIb, IV	ECOG 0–1	Cbdca (AUC 5) + S1 (80 mg/m^2^ [d 1–14 po bid]) Cbdca (AUC 6) + Ptx (200 mg/m^2^) [S]	564	64
Ouyang 2018	China	III	PFS	NSCLC	2	IIIb, IV, Rec	ECOG 0–2	Cddp (30 mg/m^2^ d2,4) + Vnr (25 mg/m^2^ d1,8) + Dulanermin (75 μg/kg) Cddp (30 mg/m^2^ d2,4) + Vnr (25 mg/m^2^ d1,8) [M][S]	453	57
Paz‐Ares 2021	Germany	III	OS	NSCLC	2	IV, Rec	ECOG 0–1	Niv (360 mg q3w) + Ipi (1 mg/kg q6w) + (1 of 4 platinum doublets, 2 cycles) (1 of 4 platinum doublets, 4 cycles) [M]	535	65
Paz‐Ares 2018	Spain	III	OS, PFS	Sq	2	IV	ECOG 0–1	Cbdca (AUC 6) + Ptx (Ptx (200 mg/m^2^) or nabPtx (100 mg/m^2^ qw)) + Pemb (200 mg) Cbdca (AUC 6) + Ptx (Ptx(200 mg/m^2^) or nabPtx (100 mg/m^2^ qw)) [M][S]	207	65
Ramalingam 2017_Sq	USA	II	PFS	Sq	2	IV	ECOG 0–1	Cbdca (AUC 6) + Ptx (200 mg/m^2^) + Veliparib (120 mg) Cbdca (AUC 6) + Ptx (200 mg/m^2^) [M][S]	76	63
Rizvi 2020	USA	III	OS, PFS	NSCLC	3	IV	ECOG 0–1	Dur (20 mg/kg) Dur 20 mg/kg + Trml (1 mg/kg) 1 of 5 platinum doublets [M]	644	65
Scagliotti 2002	Italy	III	NS	NSCLC	3	IIIb, IV, Rec	ECOG 0–2	Cddp (75 mg/m^2^) + Gem (1250 mg/m^2^ [d 1,8]) Cbdca (AUC 6) + Ptx (225 mg/m^2^) Cddp (100 mg/m^2^) + Vnr (25 mg/m^2^ [weekly]) [S]	612	63
Schiller 2002	USA	NS	OS	NSCLC	4	IIIb, IV, Rec	ECOG 0–2	(Cddp (75 mg/m^2^) or Cbdca (AUC 6)) + Ptx (135 or 225 mg/m^2^) Cddp (75 mg/m^2^) + Gem (1000 mg/m^2^ [d 1,8,15]) Cddp (75 mg/m^2^) + Dtx (75 mg/m^2^) [S]	1207	63
Schmid_2017	UK	II	PFS	Sq	2	IIIb, IV	Not available	Cbdca (AUC5) + Gem (1250 mg/m^2^) + Apatrosen (600 or 400 mg) Cbdca (AUC5) + Gem (1250 mg/m^2^) [M][S]	86	Not available
Shukuya 2015	Japan	III	OS	Sq	2	IIIb, IV, Rec	ECOG 0–1	Cdgp (100 mg/m^2^) + Dtx (60 mg/m^2^) Cddp (80 mg/m^2^) + Dtx (60 mg/m^2^) [S]	355	64
Smit 2003	Netherlands	III	OS	NSCLC	2†	IIIb, IV	ECOG 0–2	Cddp (80 mg/m^2^) + Ptx (175 mg/m^2^) Cddp (80 mg/m^2^) + Gem (1250 mg/m^2^ [d 1,8]) [S]	319	57
Spigel 2017	USA	II	RR	Sq	2	IV	ECOG 0–1	Cbdca (AUC 6) + nabPtx (200 mg/m^2^) + Nctm (800 mg) Cbdca (AUC 6) + nabPtx (200 mg/m^2^) [M][S]	167	66
Tan 2009	Singapore	III	TTF	NSCLC	2	IIIb, IV, Rec	KPS ≥80%	Cddp (80 mg/m^2^) + Vnr (30 (d1), 80 (d 8 po) mg/m^2^) Cddp (75 mg/m^2^) + Dtx (75 mg/m^2^) [S]	390	61
Thatcher 2015	UK	III	OS	Sq	2	IV	ECOG 0–2	Cddp (75 mg/m^2^) + Gem (1250 mg/m^2^) + Nctm (800 mg) Cddp (75 mg/m^2^) + Gem (1250 mg/m^2^) [M][S]	1093	62
Thomas 2006	France	II	RR	NSCLC	2	IIIb, IV	ECOG 0–2	Cbdca (AUC 6) + Gem (1250 mg/m^2^ [d 1,8]) Cddp (80 mg/m^2^) + Vnr (30 mg/m^2^ [weekly]) [S]	100	58
Thomas S 2017	USA	II	PFS	Sq	2	IV	ECOG 0–2	(Cddp [75 mg/m^2^] or Cbdca [AUC6]) + Gem (100 mg/m^2^) + Ram (10 mg/kg) (Cddp [75 mg/m^2^] or Cbdca [AUC6]) + Gem (100 mg/m^2^) [M][S]	140	early 60s
Treat 2010	USA	III	OS	NSCLC	2†	IIIb, IV, Rec	ECOG 0–2	Cbdca (AUC 5.5) + Gem (1000 mg/m^2^ [d 1,8]) Cbdca (AUC 6) + Ptx (225 mg/m^2^) [S]	758	64
Wang 2019	China	II	RR	Sq	2	III, IV	ECOG 0–1	Cbdca (AUC 5) + nabPtx (135 mg/m^2^ d1,8) Cbdca (AUC 5) + Gem (1250 mg/m^2^) [S]	127	59
Watanabe 2019	Japan	Ib/II	OS	Sq	2	IV	ECOG 0–1	Cbdca (75 mg/m^2^) + Gem (1000 or 1250 mg/m^2^) + Nctm (800 mg d1,8) Cbdca (75 mg/m^2^) + Gem (1000 or 1250 mg/m^2^) [M][S]	183	66
Wheatley 2019	USA	II	PFS	NSCLC	2	IIIb, IV	ECOG 0–1	Cbdca (AUC 6) + Ptx (200 mg/m^2^) + MEDI‐575 (25 mg/m^2^) Cbdca (AUC 6) + Ptx (200 mg/m^2^) [M][S]	81	Not available
Wu 2021	China	III	OS	NSCLC	2	IIIb, IV, Rec	ECOG 0–1	Pemb (200 mg) Cbdca (AUC 5–6) + (Ptx 200 mg/m^2^ or Pemt 500 mg/m^2^) [M]	116	62
Yang 2012	China	NS	RR	NSCLC	2	IIIb, IV	ECOG 0–2	Cdgp (80 mg/m^2^) + Gem (1250 mg/m^2^ [d 1,8]) Cbdca (AUC 5) + Gem (1250 mg/m^2^ [d 1,8]) [S]	62	57

*Note*: Study: First author, publication year, specific study name if available are presented. Patients: numbers of patients randomized for evaluated arms. Median age: when median age (years) is not available, average age (years) is presented instead. Updated: Updated data that were published later were available.

Abbreviations: NS, not specified; OS, overall survival; PFS, progression‐free survival; QOL, quality of life; RR, response rate; DCR, disease control rate; TTP, time to progression; AE, adverse event; Non‐inf; primary outcome was evaluated by non‐inferiority analysis; NSCLC, non‐small cell lung cancer; Sq, squamous carcinoma; Rec, recurrent; ECOG, Eastern Cooperative Oncology Group performance status; KPS, Karnofsky performance status; Cddp, cisplatin; Cbdca, carboplatin; Cdgp, nedaplatin; Ptx, paclitaxel; nabPtx; nanoparticle albumin‐bound Ptx; Dtx, docetaxel; Vnr, vinorelbine; Pemt, pemetrexed; Gem, gemcitabine; Cpt‐11, irinotecan; S1, tegafur gimeracil oteracil; Pemb, pembrolizumab; Niv, nivolumab; Ipi, ipilimumab; Dur, durvalumab; Trml, tremelimumab; Atz, atezolizumab; Ram, ramucirumab; Nctm, necitumumab; d, day; po, oral administration; Bid, twice daily; [M], study incorporated in main model; [S], study incorporated in separate model.

^†^ 3 > 2(excluded).

^‡^ 4 > 2(excluded).

### Efficacy analysis

**FIGURE 2:**
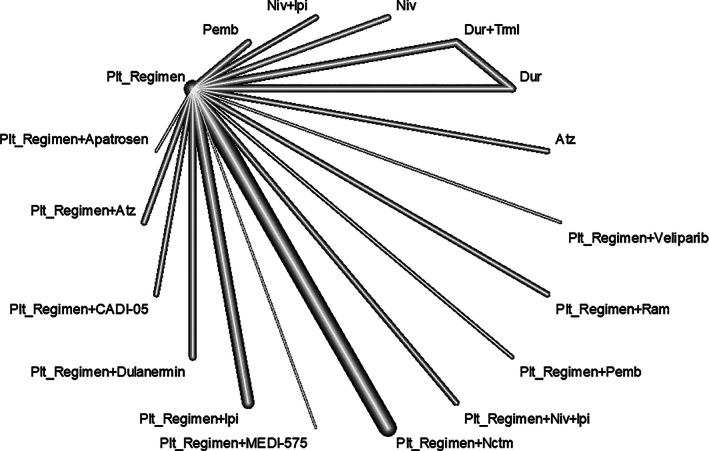
Network diagram for the primary endpoint, hazard ratio for overall survival. Addiction model, whole network level (I^2^ = 0%, *p* = 0.3943)

**FIGURE 3:**
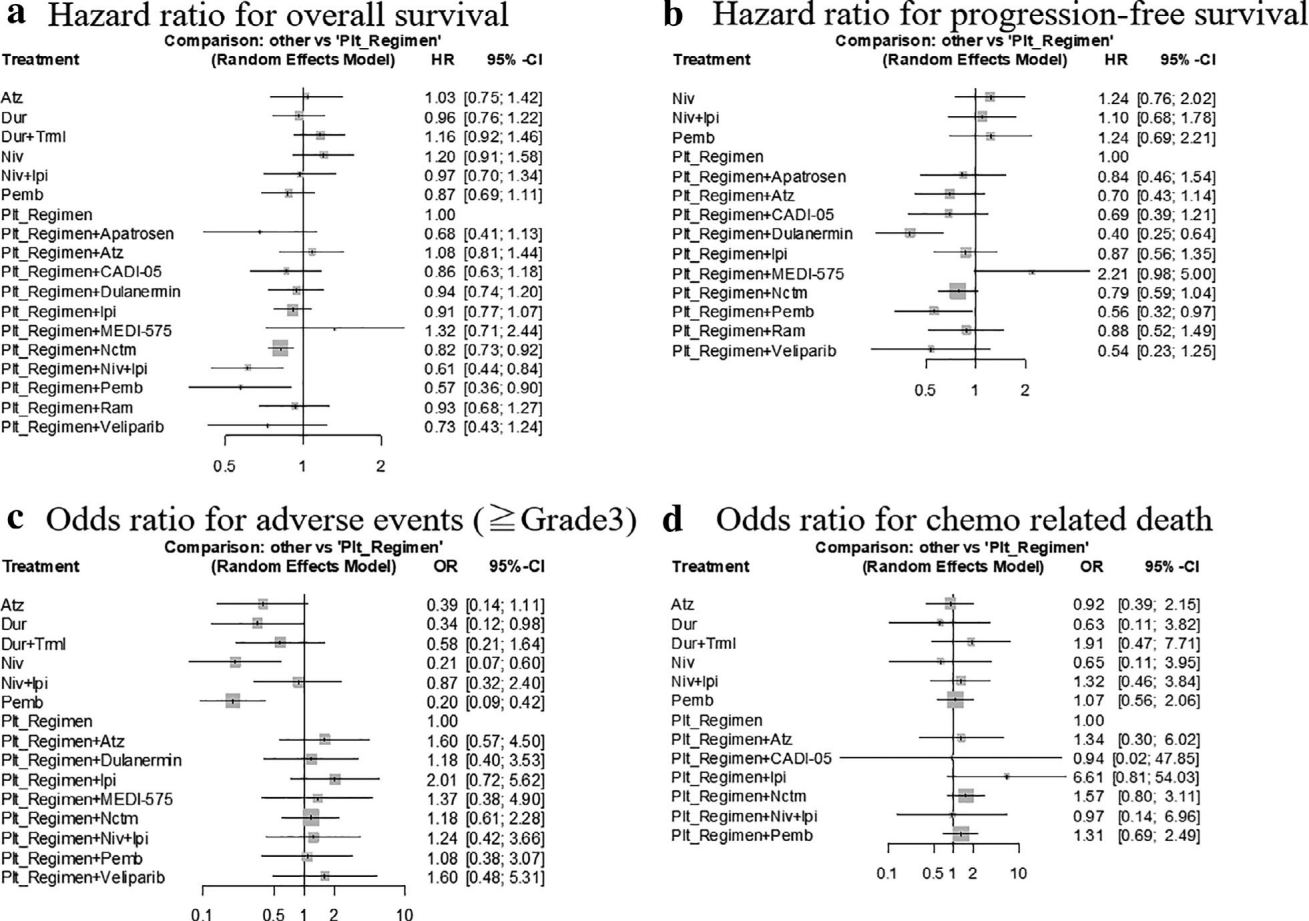
Forest plots for primary and secondary outcomes in main model. (a) Hazard ratio for overall survival (b) Hazard ratio for progression‐free survival (c) Odds ratio for adverse events (> Grade 3) (d) Odds ratio for chemo‐related death. Atz, atezolizumab; Cl, confidence interval; Dur, durvalumab; HR, hazard ratio; Ipi, ipilimumab; Nctm, necitumumab; Niv, nivolumab; OR, odds ratios; Pemb, pembrolizumab; Plt, platinum regimen; Ram, ramucirumab; Trml, tremelimumab

The hazard ratios of OS (HRos) were evaluated in 19 studies with 6785 total patients (Table 1). In the main model, the HRos ranged from 0.57 to 1.32 with a median of 0.94. There was no inconsistency between the Q statistics and the test for heterogeneity at any level (whole network level I^2^ = 0%; total *p* = 0.394; within designs, *p* = 0.394) (Figures 2 and S1). The targeted treatments were clustered in the same node. The platinum regimen + Pemb yielded the best OS benefit compared to chemotherapy (HR = 0.57, 95% CI = 0.36–0.90, *p* = 0.016), followed by the platinum regimen + nivolumab (Niv) + ipilimumab (Ipi) (HR = 0.61, 95% CI = 0.44–0.84, *p* = 0.003), and the platinum regimen + necitumumab (Nctm) (HR = 0.82, 95% CI = 0.73–0.92, *p* < 0.001) (Figure 3(a)). Atezolizumab (Atz) was not statistically different from the platinum regimen (HR = 1.08, 95% CI = 0.81–1.44, *p* = 0.60). The additional analysis including only studies in which PD‐L1 was explicitly mentioned was conducted. The results did not conflict with the main analysis (Figure S2). In the separate model, HRos of the platinum regimen + Ptx + Pemb (HR = 0.57, 95% CI = 0.36–0.90, *p* = 0.016) ranked first. The effect of this regimen was significantly different between the separate models (Figure S3).

Regarding PFS, the platinum regimen + Pemb regimen showed a significantly lower hazard ratio of progression‐free survival (HRpfs) than the platinum regimen alone (HR = 0.56, 95% CI = 0.32–0.97, *p* = 0.040). The platinum regimen + dulanermin regimen showed the best PFS (HR = 0.40, 95% CI = 0.25–0.64, *p* < 0.001) (Figure 3(b)).

### Safety analysis

Safety was considered in regard to grade III adverse events and chemotherapy‐related deaths. Pemb had a significantly lower risk of grade III adverse events than did the platinum regimen (OR = 0.20, 95% CI = 0.09–0.42, *p* < 0.001), followed by Niv and durvalumab (Figure 3(c),(d)). The addition of ICI to the platinum regimen did not result in a statistically higher risk compared to standard chemotherapy. The regimens were not significantly different for chemotherapy‐related deaths.

## DISCUSSION

We conducted a network meta‐analysis to compare regimens for chemo‐naïve incurable squamous lung cancer with a PD‐L1 TPS of 1%–49%. In addition to cytotoxic regimens and ICIs, molecularly targeted therapies were also integrated. The analysis showed that the combination of platinum regimen and ICIs was the best regimen in terms of OS and PFS, without considerably increasing the risk of adverse events. The high statistical power acquired by aggregating RCTs increases the certainty and accuracy of the results.

The platinum regimen is reported to enhance the activity of immunotherapy even though the tumor microenvironment is non‐immunogenic.^25^ The death of cancer cells triggers phagocytosis and promotes the maturation of dendritic cells, leading to tumor eradication.^26^ Therefore, combination therapy is reasonable. The main models in our study indicated that HRos of the platinum regimen + Pemb significantly decreased when compared with those of the platinum regimen (HR = 0.57, 95% CI = 0.36–0.90); this trend was followed by HRpfs of the platinum regimen + Pemb (HR = 0.56, 95% CI = 0.32–0.97) (Figure 3(a),(b)). ICI monotherapy has shown great efficacy in some patients, leading to long‐term responses.^27^ Because the OS curve of Niv + Ipi crossed that of chemotherapy in the early stage of CheckMate 227,^16^ patients receiving ICI monotherapy do not receive the benefits that come with early disease control. When chemotherapy is combined with ICIs, the possibility of rapidly controlling the initial disease increases. There is concern about the adverse effects of combination therapy with ICIs. In our analysis, the ORae of the platinum regimen + Pemb (OR = 1.08, 95% CI = 0.38–3.07) and that of the platinum regimen + Niv + Ipi (OR = 1.24, 95% CI = 0.42–3.66) were not significantly higher than that of the platinum regimen alone (Figure 3(c)). ICI monotherapy, for example, Pemb (ORae = 0.20, 95% CI = 0.09–0.42), has also shown to be safer than the platinum regimens. However, the HRos of ICI monotherapy were not statistically different from those of the platinum regimen. Therefore, we recommend combination therapy rather than ICI monotherapy for patients with a PD‐L1 TPS of 1%–49%. ICI monotherapy can be used when patients are intolerant to cytotoxic regimens.

In our study, combination therapies with drug exclusive ICIs were analyzed. The platinum regimen + Nctm was better than the platinum regimen alone in terms of OS (HR = 0.82, 95% CI = 0.73–0.92) (Figure 3(a)). In the separate model, the platinum regimen + gemcitabine + Nctm indicated the same result (HR = 0.79, 95% CI = 0.69–0.92) (Figure S3). Nctm is a monoclonal antibody against EGFR and has been reported to be effective in patients with high EGFR or squamous cancer scores. Adverse events such as skin rash, venous thromboembolism, and eye disorders should be noted.^28^

For squamous lung cancer with PD‐L1 TPS of 1%–49%, platinum + Atz is recommended by the guidelines of the American Society of Clinical Oncology, the National Comprehensive Cancer Network, and the European Society for Medical Oncology. However, the results of Impower 130,^29^ which compared platinum + Ptx + bevacizumab + Atz and platinum + Ptx + bevacizumab against squamous cancer, did not show any effect on OS. In our study, Figure 3(a),(b) indicated that HRos (HR = 1.08, 95% CI = 0.81–1.44) and HRpfs (HR = 0.70, 95% CI = 0.43–1.14) of the platinum regimen + Atz were not significantly different from those of the platinum regimen alone.

Our analysis had some limitations. First, although there were differences in the drug, amount, and period, the platinum regimen in the main model was identified to be the same. Complementarily, we conducted a separate model, and the result was similar to that of the main model. Second, according to the Cochrane tool criteria, all incorporated papers had a high risk of bias. Unfortunately, it is difficult to conduct a double‐blind study without a sponsor. We believe that these factors did not significantly reduce the reliability of this study.

In summary, we performed a systematic review and network meta‐analysis of patients with squamous NSCLC with a PD‐L1 TPS of 1%–49%. For the 16 391 patients diagnosed with NSCLC and part of 48 RCTs, the platinum regimen + Pemb and the platinum regimen + Niv + Ipi were considered appropriate first‐line agents for treating squamous NSCLC with low PD‐L1.

## CONFLICT OF INTEREST

All authors have completed the ICMJE uniform disclosure form. N.H. has received personal fee from Taiho Pharmaceutical and a research grant from Taiho Pharmaceutical outside of the work. K.W. has received personal fees from AstraZeneca, Ono Pharmaceutical, and Boehringer Ingelheim outside of the work. Y.H. has received personal fees from AstraZeneca and Boehringer Ingelheim outside of the work. N.K. has received personal fees from Chugai Pharmaceutical, AstraZeneca, Boehringer Ingelheim, Sanofi, Ono Pharmaceutical, MSD, Bristol Myers Squibb, Eli Lilly, and Kyowa Kirin; and research grants from Chugai Pharmaceutical, Boehringer Ingelheim, MSD, Eli Lilly, Kyowa Kirin, Daiichi Sankyo, and Pfizer outside of the work. T.K. has received personal fees from Chugai Pharmaceutical, AstraZeneca, Bristol Myers Squibb, Eli Lilly, Taiho Pharmaceutical, Chugai Pharmaceutical, Daiichi Sankyo, Sanofi, and Pfizer; and research grants from MSD, Chugai Pharmaceutical, Eli Lilly, Taiho Pharmaceutical, Chugai Pharmaceutical, Daiichi Sankyo, Pfizer, and Shionogi outside of the work. The other authors have no conflicts of interest to declare.

## Supporting information


**Appendix S1** Supporting Information

## References

[1] Jemal A , Bray F , Center MM . Global cancer statistics. CA Cancer J Clin. 2011;61:69–90. 21296855 10.3322/caac.20107

[2] Rojas JJ , Sampath P , Hou W , Thorne SH . Defining effective combinations of immune checkpoint blockade and oncolytic virotherapy. Clin Cancer Res. 2015;21:5543–51. 26187615 10.1158/1078-0432.CCR-14-2009 PMC4681662

[3] Massarelli E , Papadimitrakopoulou V , Welsh J , Tang C , Tsao AS . Immunotherapy in lung cancer. Transl Lung Cancer Res. 2014;3:53–63. 25806281 10.3978/j.issn.2218-6751.2014.01.01 PMC4367607

[4] Akamatsu H , Ninomiya K , Kenmotsu H , Morise M , Daga H , Goto Y , et al. The Japanese Lung Cancer Society guideline for non‐small cell lung cancer, stage IV. Int J Clin Oncol. 2019;24:731–70. 31049758 10.1007/s10147-019-01431-z PMC6545178

[5] Hanna NH , Schneider BJ , Temin S , Baker S Jr , Brahmer J , Ellis PM , et al. Therapy for stage IV non‐small‐cell lung cancer without driver alterations: ASCO and OH (CCO) joint guideline update. J Clin Oncol. 2020;38:1608–32. 31990617 10.1200/JCO.19.03022

[6] National Comprehensive Cancer Network . NCCN clinical practice guidelines in oncology: non‐small cell lung cancer. 2020 [cited 2021 April 14]. Available from: https://www.nccn.org

[7] European Society for Medical Oncology . Metastatic non‐small cell lung cancer: ESMO Clinical Practice Guidelines for diagnosis, treatment and follow‐up: updated version published 15. 2020 Sept [cited 2021 April 13]. Available from: https://www.esmo.org.

[8] Wang B‐Y , Huang JY , Chen HC , Lin CH , Lin SH , Hung WH , et al. The comparison between adenocarcinoma and squamous cell carcinoma in lung cancer patients. J Cancer Res Clin Oncol. 2020;146:43–52. 31705294 10.1007/s00432-019-03079-8 PMC11804334

[9] Rotow J , Bivona TG . Understanding and targeting resistance mechanisms in NSCLC. Nat Rev Cancer. 2017;17:637–58. 29068003 10.1038/nrc.2017.84

[10] Horita N , Nagashima A , Nakashima K , Shibata Y , Ito K , Goto A , et al. The best platinum regimens for chemo‐naïve incurable non‐small cell lung cancer: network meta‐analysis. Sci Rep. 2017;7:13185. 29030633 10.1038/s41598-017-13724-2 PMC5640659

[11] Hutton B , Salanti G , Caldwell DM , Chaimani A , Schmid CH , Cameron C , et al. The PRISMA extension statement for reporting of systematic reviews incorporating network meta‐analyses of health care interventions: checklist and explanations. Ann Intern Med. 2015;162:777–84. 26030634 10.7326/M14-2385

[12] University Hospital Medical Information Network (UMIN) Center . [cited 2020 Oct 15]. Available from: https://www.umin.ac.jp/ctr/ctr_regist.htm

[13] Hotta K , Matsuo K , Ueoka H , Kiura K , Tabata M , Tanimoto M . Meta‐analysis of randomized clinical trials comparing cisplatin to carboplatin in patients with advanced non‐small‐cell lung cancer. J Clin Oncol. 2004;22:3852–9. 15326195 10.1200/JCO.2004.02.109

[14] Griesinger F , Korol EE , Kayaniyil S , Varol N , Ebner T , Goring SM . Efficacy and safety of first‐line carboplatin‐versus cisplatin‐based chemotherapy for non‐small cell lung cancer: a meta‐analysis. Lung Cancer. 2019;135:196–204. 31446995 10.1016/j.lungcan.2019.07.010

[15] Gandhi L , Rodríguez‐Abreu D , Gadgeel S , Esteban E , Felip E , de Angelis F , et al. Pembrolizumab plus chemotherapy in metastatic non‐small‐cell lung cancer. N Engl J Med. 2018;378:2078–92. 29658856 10.1056/NEJMoa1801005

[16] Hellmann MD , Paz‐Ares L , Bernabe CR , Zurawski B , Kim SW , Carcereny Costa E , et al. Nivolumab plus ipilimumab in advanced non‐small‐cell lung cancer. N Engl J Med. 2019;381:2020–31. 31562796 10.1056/NEJMoa1910231

[17] Paz‐Ares L , Luft A , Vicente A , Tafreshi A , Gümüş M , Mazières J , et al. Pembrolizumab plus chemotherapy for squamous non‐small‐cell lung cancer. N Engl J Med. 2018;379:2040–51. 30280635 10.1056/NEJMoa1810865

[18] Paver EC , Cooper WA , Colebatch AJ , Ferguson PM , Hill SK , Lum T , et al. Programmed death ligand‐1 (PD‐L1) as a predictive marker for immunotherapy in solid tumours: a guide to immunohistochemistry implementation and interpretation. Pathology. 2021;53:141–56. 33388161 10.1016/j.pathol.2020.10.007

[19] Higgins JP , Altman DG , Gøtzsche PC , Jüni P , Moher D , Oxman AD , et al. The Cochrane Collaboration's tool for assessing risk of bias in randomised trials. BMJ. 2011;343:d5928. 22008217 10.1136/bmj.d5928 PMC3196245

[20] Atkinson TM , Ryan SJ , Bennett AV , Stover AM , Saracino RM , Rogak LJ , et al. The association between clinician‐based common terminology criteria for adverse events (CTCAE) and patient‐reported outcomes (PRO): a systematic review. Support Care Cancer. 2016;24:3669–76. 27260018 10.1007/s00520-016-3297-9 PMC4919215

[21] Eisenhauer EA , Therasse P , Bogaerts J , Schwartz LH , Sargent D , Ford R , et al. New response evaluation criteria in solid tumours: revised RECIST guideline (version 1.1). Eur J Cancer. 2009;45:228–47. 19097774 10.1016/j.ejca.2008.10.026

[22] Parmar MK , Torri V , Stewart L . Extracting summary statistics to perform meta‐analyses of the published literature for survival endpoints. Stat Med. 1998;17:2815–34. 9921604 10.1002/(sici)1097-0258(19981230)17:24<2815::aid-sim110>3.0.co;2-8

[23] Salanti G . Indirect and mixed‐treatment comparison, network, or multiple‐treatments meta‐analysis: many names, many benefits, many concerns for the next generation evidence synthesis tool. Res Synth Methods. 2012;3:80–97. 26062083 10.1002/jrsm.1037

[24] Rucker G. 2016. Package “netmeta”: network Meta‐Analysis using frequentist Methods. [cited 2021 April 14]. Available from: https://cran.r-project.org/web/packages/netmeta/netmeta.pdf#search=

[25] Pfirschke C , Engblom C , Rickelt S , Cortez‐Retamozo V , Garris C , Pucci F , et al. Immunogenic chemotherapy sensitizes tumors to checkpoint blockade therapy. Immunity. 2016;44:343–54. 26872698 10.1016/j.immuni.2015.11.024 PMC4758865

[26] Bracci L , Schiavoni G , Sistigu A , Belardelli F . Immune‐based mechanisms of cytotoxic chemotherapy: implications for the design of novel and rationale‐based combined treatments against cancer. Cell Death Differ. 2014;21:15–25. 23787994 10.1038/cdd.2013.67 PMC3857622

[27] Frigola J , Navarro A , Carbonell C , Callejo A , Iranzo P , Cedrés S , et al. Molecular profiling of long‐term responders to immune checkpoint inhibitors in advanced non‐small cell lung cancer. Mol Oncol. 2021;15:887–900. 33342055 10.1002/1878-0261.12891 PMC8024716

[28] Wang L , Liao C , Li M , Zhang S , Yi F , Wei Y , et al. Necitumumab plus platinum‐based chemotherapy versus chemotherapy alone as first‐line treatment for stage IV non‐small cell lung cancer: a meta‐analysis based on randomized controlled trials. Ann Palliat Med. 2021;10:1154–66. 32954751 10.21037/apm-19-365

[29] Jotte R , Cappuzzo F , Vynnychenko I , Stroyakovskiy D , Rodríguez‐Abreu D , Hussein M , et al. Atezolizumab in combination with carboplatin and nab‐paclitaxel in advanced squamous NSCLC (IMpower131): results from a randomized phase III trial. J Thorac Oncol. 2020;15:1351–60. 32302702 10.1016/j.jtho.2020.03.028

