# Supplementary material for: Best regimens for treating chemo‐naïve incurable squamous non‐small cell lung cancer with a programmed death‐ligand 1 tumor proportion score of 1%–49%: A network meta‐analysis
Source: Thorac Cancer. 2021 Nov 17;13(1):84–94. doi: 10.1111/1759-7714.14229 (PMC8720615; doi:10.1111/1759-7714.14229)
Supplement: Supplementary file 1 — Appendix S1 Supporting Information [file TCA-13-84-s001.docx]

The best regimens for chemo-naïve incurable squamous non-small cell lung cancer with a programmed death-ligand 1, tumor proportion score 1%-49%: A network meta-analysis

**<Supplementary file>**

Authors:

Nobuhiko Fukuda, Nobuyuki Horita, Ho Namkoong, Ayami Kaneko, Kouhei Somekawa, Youichi Tagami, Keisuke Watanabe, Yu Hara, Nobuaki Kobayashi and Takeshi Kaneko

**Supplementary Text 1.** The amendments to information in the protocol.

・Odds ratio of objective response rate was not evaluated.

・The populations with tumor proportion score 0% were excluded.

**Supplementary Text 2.** Search formulas.

*MEDLINE*

(non-small OR squamous OR adenocarcinoma OR non-squamous OR NSCLC) AND (lung cancer OR lung carcinoma OR lung malignancy OR lung tumor OR NSCLC) AND (advanced OR metastasis OR recurrent OR recurrence OR inoperable OR relapsed OR incurable OR stage 3 OR stage 3a OR stage 3b OR stage Ⅲ OR stage Ⅲa OR stage Ⅲb OR stage 4 OR stage 4a OR stage 4b OR stage Ⅳ OR stage Ⅳa OR stage Ⅳb) AND (naïve OR untreated OR chemo naïve OR chemo-naïve OR non-treated OR nontreated OR first-line OR front-line OR initial treatment OR “previously not treated”) AND (randomised[title] OR randomized[title] OR randomly OR phase 3[title] OR phase Ⅲ[title] OR RCT[title] OR (nejm AND (randomized OR randomly OR phase 3 OR phase Ⅲ OR RCT))).

**Supplementary Text 3.** Characteristics of the included studies.

The included studies were reported in a variety of countries worldwide. The United States of America had the most included studies (14 studies). The articles were published between 2000 and 2021. Among 48 reports, 28 were phase III studies, 25 evaluated OS as the primary endpoint, 28 included ECOG 0-1 cases. We regarded 3 studies as three-arm studies, 1 study as a four-arm study, and the other 44 as two-arm studies.

**Supplementary Figure 1.**

Network diagram for the primary endpoint, hazard ration for overall survival.

Separate model, Whole network level (I^2^ = 0%, Total; P = 0.8544, Within designs; P = 0.8791, Between designs; P = 0.5526).

Plt, platinum regimen; Ptx, paclitaxel; Gem, gemcitabine; Ram, ramucirumab; Nctm, necitumumab; Dtx, docetaxel; Cpt11, irinotecan; Pemb, pembrolizumab; Cdgp, nedaplatin; Vnr, vinorelbine; S1, tegafur gimeracil oteracil; Ipi, ipilimumab; Atz, atezolizumab

Supplementary Figure 2.

Forest plots for primary outcomes (hazard ratio for overall survival) in main model that included only studies in which PD-L1 was explicitly mentioned.

Plt, platinum regimen; Pemb, pembrolizumab; Niv, nivolumab; Ipi, ipilimumab; Dur, durvalumab; Trml, Tremelimumab; Atz, atezolizumab; HR, hazard ratio; Cl, confidence interval.

**Supplementary Figure 3.**

Forest plots for primary and secondary outcomes in separate model.

A) Hazard ratio for overall survival

B) Hazard ratio for progression-free survival


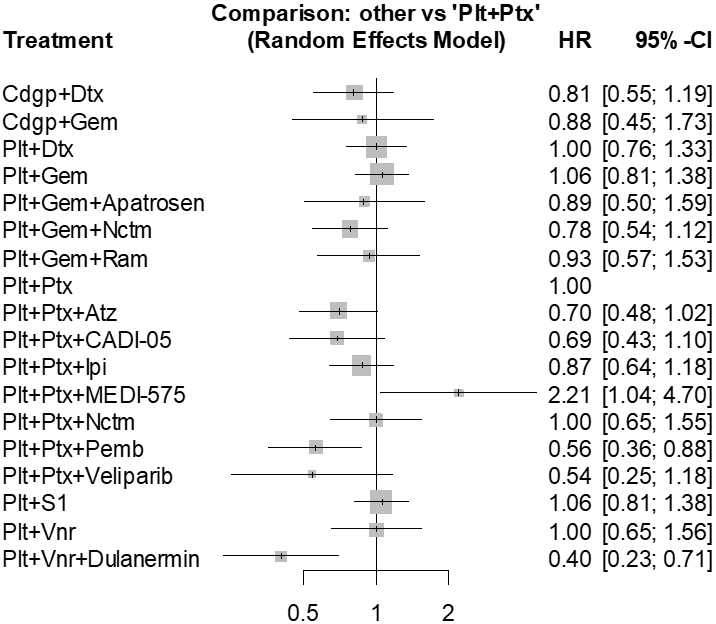


C) Odds ratio for adverse events (≧Grade3)

D) Odds ratio for chemo related death


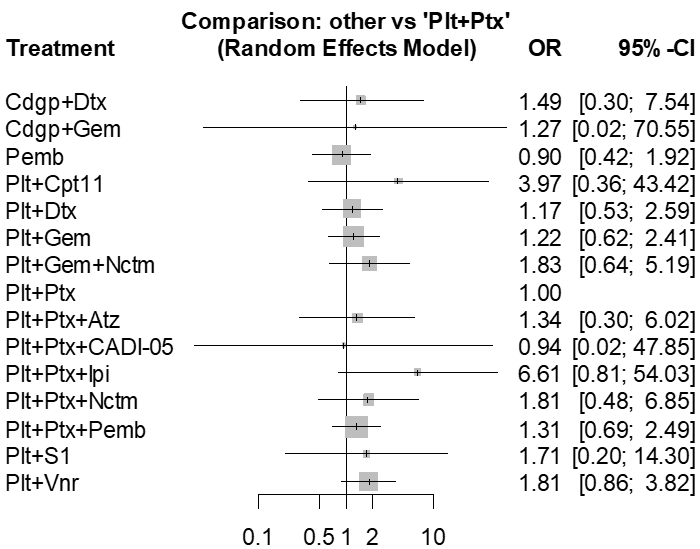


Plt, platinum regimen; Ptx, paclitaxel; Gem, gemcitabine; Ram, ramucirumab; Nctm, necitumumab; Dtx, docetaxel; Cpt11, irinotecan; Pemb, pembrolizumab; Cdgp, nedaplatin; Vnr, vinorelbine; S1, tegafur gimeracil oteracil; Ipi, ipilimumab; Atz, atezolizumabHR, hazard ratio; OR, Odds ratio; Cl, confidence interval

**Supplementary Table.** The Cochrane Risk of Bias evaluation sheet.

|  | selection bias | performance bias | detection bias | attrition bias | reporting bias | other bias |
| --- | --- | --- | --- | --- | --- | --- |
| Belani_2017 | Low | High | Low | Low | Low | Low |
| Carbone_2017 | Low | High | Low | Low | Low | High |
| Chang_2008 | Unclear | High | Low | Low | Low | Low |
| Chen_2007 | Unclear | High | Low | Low | Low | Low |
| Chen_2004 | Unclear | High | Low | Low | High | Low |
| Comella_2000 | Low | High | Low | Low | Low | Low |
| Douillard_2005 | Unclear | High | Low | Low | Low | High |
| Edelman_2004 | Low | High | Low | Low | Low | Low |
| Fossella_2003 | Low | High | Low | Low | Low | High |
| Gebbia_2010 | Unclear | High | Unclear | Low | Low | Low |
| Gebbia_2003 | Low | High | Low | Low | Low | Low |
| Govindan_2017 | Low | Low | Low | Low | Low | High |
| Grossi_2018 | Unclear | High | Unclear | Unclear | Low | Unclear |
| Harada_2019 | Unclear | High | Unclear | low | Low | Unclear |
| Helbekkmo_2007 | Low | High | Low | Low | Low | Low |
| Hellmann_2019 | Low | High | Low | Low | Low | High |
| Herbst_2020 | Low | High | Low | Low | Low | High |
| Jotte_2020 | Low | High | Low | Low | Low | High |
| Kawahara_2013 | Low | High | Low | Low | Low | Low |
| Khodadad_2014 | Unclear | High | Low | Low | Low | High |
| Kubota_2015 | Low | High | Low | Low | Low | High |
| Lu_2018 | Low | High | Unclear | Low | Low | High |
| Martoni_2005 | Unclear | High | Unclear | Low | Low | Low |
| Minami_2013 | Unclear | High | Low | Low | Low | Low |
| Mok_2019_sq | Low | High | Low | Low | Low | High |
| Ohe_2007 | Low | High | Low | Low | Low | High |
| Okamoto_2010 | Low | High | Low | Low | Low | High |
| Ouyang_2018 | Low | Low | Low | Low | Low | High |
| Paz-Ares_2021 | Unclear | High | Low | Low | Low | High |
| Paz-Ares_2018 | Low | Low | Low | Low | Low | High |
| Ramalingam_2017_Sq | Low | Low | Low | Low | Low | High |
| Rizvi_2020 | Low | High | Low | Low | Low | High |
| Scagliotti_2002 | Low | High | Low | Low | High | High |
| Schiller_2002 | Unclear | High | Low | Low | Low | Low |
| Schmid_2017 | Unclear | High | Low | Unclear | Low | High |
| Shukuya_2015 | Low | High | Low | Low | Low | High |
| Smit_2003 | Low | High | Low | Low | Low | High |
| Spigel_2017 | Low | High | Low | Low | Low | High |
| Tan_2009 | Low | High | Low | Low | Low | Low |
| Thatcher_2015 | Low | High | Low | Low | Low | High |
| Thomas_2006 | Unclear | High | Low | Low | Low | High |
| ThomasS_2017 | Unclear | High | Low | Unclear | Low | High |
| Treat_2010 | Unclear | High | Low | Low | Low | High |
| Wang_2019 | Unclear | High | Low | Low | Low | High |
| Watanabe_2019 | Low | High | Low | Low | Low | High |
| Wheatley_2019 | Low | High | Low | Low | Low | High |
| Wu_2021 | Low | High | Low | Low | Low | High |
| Yang_2012 | Unclear | High | Low | High | Low | Low |

One study had a high risk of selection bias due to randomization using an envelope method, 44 studies had a high risk of performance bias due to a non-blinded study design, 1 study had a high risk of attrition bias because 21% of the randomized patients did not receive the assigned regimen, and 2 studies had a high risk of reporting bias because the primary endpoint was not specified. Thirty-three studies were marked as having a high risk of other bias for potential conflicts of interest because the studies were directly funded or advised by pharmaceutical companies.

**Supplementary References.** List of included studies.

1: Belani CP, Chakraborty BC, Modi RI et al. A randomized trial of TLR-2 agonist CADI-05 targeting desmocollin-3 for advanced non-small-cell lung cancer. Ann Oncol 2017; 28: 298-304. doi:10.1093/annonc/mdw608.

2: Carbone DP, Reck M, Paz-Ares L et al. First-Line Nivolumab in Stage IV or Recurrent Non–Small-Cell Lung Cancer. N Engl Med 2017; 376: 2415-2426. doi: 10.1056/NEJMoa1613493.

3: Chang JW-CT, Tsao T, Yang C et al. A randomized study of gemcitabine plus cisplatin and vinorelbine plus cisplatin in patients with advanced non-small-cell lung cancer. Chang Gung Med J 2008; 31: 559-566.

4: Chen YM, Perng RP, Shin JF et al. A randomised phase II study of weekly paclitaxel or vinorelbine in combination with cisplatin against inoperable non-small-cell lung cancer previously untreated. Br J Cancer 2004; 90: 359-365. doi: 10.1038/sj.bjc.6601526.

5: Chen YM, Perng RP, Shin JF et al. A randomized phase II study of docetaxel or vinorelbine in combination with cisplatin against inoperable, chemo-naive non-small-cell lung cancer in Taiwan. Lung cancer 2007; 56: 363-369. doi: 10.1016/j.lungcan.2007.01.011.

6: Comella P, Frasci G, Panza N et al. Randomized trial comparing cisplatin, gemcitabine, and vinorelbine with either cisplatin and gemcitabine or cisplatin and vinorelbine in advanced non-small-cell lung cancer: interim analysis of a phase III trial of the Southern Italy Cooperative Oncology Group. J Clin Oncol 2000; 18: 1451-1457. doi: 10.1200/JCO.2000.18.7.1451.

7: Douillard JY, Gervais R, Dabouis G et al. Sequential two-line strategy for stage IV non-small-cell lung cancer: docetaxel-cisplatin versus vinorelbine-cisplatin followed by cross-over to single-agent docetaxel or vinorelbine at progression: final results of a randomised phase II study. Ann Oncol 2005; 16: 81-89. doi: 10.1093/annonc/mdi013.

8: Edelman MJ, Clark JI, Chansky K et al. Randomized phase II trial of sequential chemotherapy in advanced non-small cell lung cancer (SWOG 9806): carboplatin/gemcitabine followed by paclitaxel or cisplatin/vinorelbine followed by docetaxel. Clin Cancer Res 2004; 10: 5022-5026. doi: 10.1158/1078-0432.CCR-04-0002.

9: Fossella F, Pereira JR, Pawel J et al. Randomized, multinational, phase III study of docetaxel plus platinum combinations versus vinorelbine plus cisplatin for advanced non-small-cell lung cancer: the TAX 326 study group. J Clin Oncol 2003; 21: 3016-3024. doi: 10.1200/JCO.2003.12.046.

10: Gebbia V, Lorusso V, Galetta D et al. First-line cisplatin with docetaxel or vinorelbine in patients with advanced non-small-cell lung cancer: a quality of life directed phase II randomized trial of Gruppo Oncologico Italia Meridionale. Lung cancer 2010; 69: 218-224. doi: 10.1016/j.lungcan.2009.10.008.

11: Gebbia V, Galetta D, Caruso M et al. Gemcitabine and cisplatin versus vinorelbine and cisplatin versus ifosfamide+gemcitabine followed by vinorelbine and cisplatin versus vinorelbine and cisplatin followed by ifosfamide and gemcitabine in stage IIIB-IV non small cell lung carcinoma: a prospective randomized phase III trial of the Gruppo Oncologico Italia Meridionale. Lung cancer 2003; 39: 179-189. doi: 10.1016/s0169-5002(02)00444-0.

12: Govindan R, Szczesna A, Ahn MJ et al. Phase III Trial of Ipilimumab Combined With Paclitaxel and Carboplatin in Advanced Squamous Non–Small-Cell Lung Cancer. J Clin Oncol 2017; 35: 3449-3457. doi: 10.1200/JCO.2016.71.7629.

13: Grossi F, Jaskiewicz P, Pichon E et al. Randomized phase II trial of oral vinorelbine (OV) and cisplatin (P) followed by maintenance with single-agent OV versus (vs) gemcitabine (GEM) and P followed by maintenance with single-agent GEM as first-line chemotherapy (CT) in locally advanced (LA) or metastatic non-small-cell lung cancer (NSCLC) patients (pts) with squamous (sq) histological type. J Clin Oncol 2018; 36: 15. doi: 10.1200/JCO.2018.36.15_suppl.e21058.

14: Harada T, Kawashima Y, Fujita Y et al. Randomized Phase II Trial of CBDCA+nab-PTX vs CDDP+GEM in Patients with Chemo-Naïve Squamous Cell Lung Cancer: NJLCG1302. Journal of Thorac Oncol 2019; 14: S357. doi: 10.1016/j.jtho.2019.08.723.

15: Helbekkmo N, Sundstrom SH, Aasebo U et al. Vinorelbine/carboplatin vs gemcitabine/carboplatin in advanced NSCLC shows similar efficacy, but different impact of toxicity. Br J Cancer 2007; 97: 283-289. doi: 10.1038/sj.bjc.6603869.

16: Hellmann L, Paz-Ares R, Bernabe CB et al. Nivolumab plus Ipilimumab in Advanced

Non–Small-Cell Lung Cancer. N Engl J Med 2019; 381: 2020-2031. doi: 10.1056/NEJMoa1910231.

17: Herbst RS, Giaccone G, Marinis F et al. Atezolizumab for First-Line Treatment

of PD-L1–Selected Patients with NSCLC. N Engl J Med 2020; 383: 1328-1339. doi: 10.1056/NEJMoa1917346.

18: Jotte R, Cappuzzo F, Vynnychenko I et al. Atezolizumab in Combination With Carboplatin and Nab-Paclitaxel in Advanced Squamous NSCLC (IMpower131): Results From a Randomized Phase III Trial. J Thorac Oncol 2020; 15: 1351-1360. doi: 10.1016/j.jtho.2020.03.028.

19: Kawahara M, Atagi S, Komuta K et al. Carboplatin plus either docetaxel or paclitaxel for Japanese patients with advanced non-small cell lung cancer. Anticancer Res 2013; 33: 4631-4637.

20: Khodadad K, Khosravi A, Esfahani-Monfared Z et al. Comparing docetaxel plus Cisplatin with Paclitaxel plus Carboplatin in chemotherapy-naive patients with advanced non-small-cell lung cancer: a single institute study. Iran J Pharm Res 2014; 13: 575-581.

21: Kubota K, Sakai H, Katakami N et al. A randomized phase III trial of oral S-1 plus cisplatin versus docetaxel plus cisplatin in Japanese patients with advanced non-small-cell lung cancer: TCOG0701 CATS trial. Ann Oncol 2015; 26: 1401-1408. doi: 10.1093/annonc/mdv190.

22: Lu S, Chen ZW, Hu CP et al. Nedaplatin Plus Docetaxel Versus Cisplatin Plus Docetaxel as First-Line Chemotherapy for Advanced Squamous Cell Carcinoma of the Lung - A Multicenter, Open-label, Randomized, Phase III Trial. J Thorac Oncol 2018; 13: 1743-1749. doi: 10.1016/j.jtho.2018.07.006.

23: Martoni A, Marino A, Sperandi F et al. Multicentre randomised phase III study comparing the same dose and schedule of cisplatin plus the same schedule of vinorelbine or gemcitabine in advanced non-small cell lung cancer. Eur J Cancer 2005; 41: 81-92. doi: 10.1016/j.ejca.2004.08.029.

24: Minami S, Kijima T, Shiroyama T et al. Randomized Phase II trial of paclitaxel and carboplatin followed by gemcitabine switch-maintenance therapy versus gemcitabine and carboplatin followed by gemcitabine continuation-maintenance therapy in previously untreated advanced non-small cell lung cancer. BMC Res Notes 2013; 6: 3. doi: 10.1186/1756-0500-6-3.

25: Mok TS, Wu Y, Kudaba I et al. Pembrolizumab versus chemotherapy for previously untreated, PD-L1-expressing, locally advanced or metastatic non-small-cell lung cancer (KEYNOTE-042): a randomised, open-label, controlled, phase 3 trial. Lancet 2019; 393: 1819-1830. doi: 10.1016/S0140-6736(18)32409-7.

26: Ohe Y, Ohashi Y, Kubota K et al. Randomized phase III study of cisplatin plus irinotecan versus carboplatin plus paclitaxel, cisplatin plus gemcitabine, and cisplatin plus vinorelbine for advanced non-small-cell lung cancer: Four-Arm Cooperative Study in Japan. Ann Oncol 2007; 18: 317-323. doi: 10.1093/annonc/mdl377.

27: Okamoto I, Yoshioka H, Morita S et al. Phase III trial comparing oral S-1 plus carboplatin with paclitaxel plus carboplatin in chemotherapy-naive patients with advanced non-small-cell lung cancer: results of a west Japan oncology group study. J Clin Oncol 2010; 28: 5240-5246. doi: 10.1200/JCO.2010.31.0326.

28: Ouyang X, Shi M, Jie F et al. Phase III study of dulanermin (recombinant human tumor necrosis factor-related apoptosis-inducing ligand/Apo2 ligand) combined with vinorelbine and cisplatin in patients with advanced non-small-cell lung cancer. Invent New Drugs 2018; 36: 315-322. doi: 10.1007/s10637-017-0536-y.

29: Paz-Ares L, Ciuleanu T-E, Cobo M et al. First-line nivolumab plus ipilimumab combined with two cycles of chemotherapy in patients with non-small-cell lung cancer (CheckMate 9LA): an international, randomised, open-label, phase 3 trial. Lancet Oncol 2021 22: 198-211. doi: 10.1016/S1470-2045(20)30641-0.

30: Paz-Ares L, Luft A, Vicente D et al. Pembrolizumab plus Chemotherapy for Squamous Non-Small-Cell Lung Cancer. N Engl J Med 2018; 379: 2040-2051. doi: 10.1056/NEJMoa1810865.

31: Ramalingam SS, Blais N, Mazieres J et al. Randomized, Placebo-Controlled, Phase II Study of Veliparib in Combination with Carboplatin and Paclitaxel for Advanced/Metastatic Non-Small Cell Lung Cancer. Clin Cancer Res 2017; 23: 1937:1944. doi: 10.1158/1078-0432.CCR-15-3069.

32: Rivzi NA, Cho BC, Reinmuth N et al. Durvalumab With or Without Tremelimumab vs Standard Chemotherapy in First-line Treatment of Metastatic Non-Small Cell Lung Cancer: The MYSTIC Phase 3 Randomized Clinical Trial. JAMA Oncol 2020; 6: 661-674. doi: 10.1001/jamaoncol.2020.0237.

33: Scagliotti GV, Marinis FD, Rinaldi M et al. Phase III randomized trial comparing three platinum-based doublets in advanced non-small-cell lung cancer. J Clin Oncol 2002; 20: 4285-4291. doi: 10.1200/JCO.2002.02.068.

34: Schiller JH, Harrington D, Belani CP et al. Comparison of four chemotherapy regimens for advanced non-small-cell lung cancer. N Engl J Med 2002; 346: 92-98. doi: 10.1056/NEJMoa011954.

35: Schmid P, Blackhall F, Muthukumar D et al. A phase II, randomised, open-label study of gemcitabine/carboplatin first-line chemotherapy in combination with or without the antisense oligonucleotide apatorsen (OGX-427) in advanced squamous cell lung cancers. Ann Oncol 2017; 28: v638. doi: 10.1093/annonc/mdx440.056.

36: Shukuya T, Yamanaka T, Seto T et al. Nedaplatin plus docetaxel versus cisplatin plus docetaxel for advanced or relapsed squamous cell carcinoma of the lung (WJOG5208L): a randomised, open-label, phase 3 trial. Lancet Oncol 2015; 16: 1630-1638. doi: 10.1016/S1470-2045(15)00305-8.

37: Smit EF, Meerbeeck JP, Lianes P et al. Three-arm randomized study of two cisplatin-based regimens and paclitaxel plus gemcitabine in advanced non-small-cell lung cancer: a phase III trial of the European Organization for Research and Treatment of Cancer Lung Cancer Group--EORTC 08975. J Clin Oncol 2003; 21: 3909-3917. doi: 10.1200/JCO.2003.03.195.

38: Spigel DR, Shipley DL, Waterhouse DM et al. A Randomized, Double-Blinded, Phase II Trial of Carboplatin and Pemetrexed with or without Apatorsen (OGX-427) in Patients with Previously Untreated Stage IV Non-Squamous-Non-Small-Cell Lung Cancer: The SPRUCE Trial. Oncologist 2019; 24: e1409-e1416. doi: 10.1634/theoncologist.2018-0518.

39: Tan EH, Rolski J, Grodzki T et al. Global Lung Oncology Branch trial 3 (GLOB3): final results of a randomised multinational phase III study alternating oral and i.v. vinorelbine plus cisplatin versus docetaxel plus cisplatin as first-line treatment of advanced non-small-cell lung cancer. Ann Oncol 2009; 20: 1249-1256. doi: 10.1093/annonc/mdn774.

40. Thatcher N, Hirsch FR, Luft AV et al. Necitumumab plus gemcitabine and cisplatin versus gemcitabine and cisplatin alone as first-line therapy in patients with stage IV squamous non-small-cell lung cancer (SQUIRE): an open-label, randomised, controlled phase 3 trial. Lancet Oncol 2015; 16: 763-774. doi: 10.1016/S1470-2045(15)00021-2.

41: Thomas P, Robinet G, Gouva S et al. Randomized multicentric phase II study of carboplatin/gemcitabine and cisplatin/vinorelbine in advanced non-small cell lung cancer GFPC 99-01 study (Groupe français de pneumo-cancérologie). Lung cancer 2006; 51: 105-114. doi: 10.1016/j.lungcan.2005.10.004.

42: Thomas S, Doebele RC, Spigel D et al. A phase 2 randomized open-label study of ramucirumab (RAM) plus first-line platinum-based chemotherapy in patients (pts) with recurrent or advanced non-small cell lung cancer (NSCLC): Final results from squamous pts. Ann Oncol 2017; 28: 42-43. doi: 10.1093/annonc/mdx085

43: Treat JA, Gonin R, Sochinski MA et al. A randomized, phase III multicenter trial of gemcitabine in combination with carboplatin or paclitaxel versus paclitaxel plus carboplatin in patients with advanced or metastatic non-small-cell lung cancer. Ann Oncol 2010; 21: 540-547. doi: 10.1093/annonc/mdp352.

44: Wang Z, Huang C, Yang JJ et al. A randomised phase II clinical trial of nab-paclitaxel and carboplatin compared with gemcitabine and carboplatin as first-line therapy in advanced squamous cell lung carcinoma (C-TONG1002). Eur J Cancer 2019; 109: 183-191. doi: 10.1016/j.ejca.2019.01.007.

45: Watanabe S, Yoshioka H, Sakai H et al. Necitumumab plus gemcitabine and cisplatin versus gemcitabine and cisplatin alone as first-line treatment for stage IV squamous non-small cell lung cancer: A phase 1b and randomized, open-label, multicenter, phase 2 trial in Japan. Lung Cancer 2019; 129: 55-62. doi: 10.1016/j.lungcan.2019.01.005.

46: Wheatley-Price P, Gadgeel S, Takahashi T et al. Phase 1b/2 Randomized Study of MEDI-575 in Combination With Carboplatin Plus Paclitaxel Versus Carboplatin Plus Paclitaxel Alone in Adult Patients With Previously Untreated Advanced Non-Small-Cell Lung Cancer. Clin Lung Cancer 2019; 20: e362-e368. doi: 10.1016/j.cllc.2018.11.012.

47: Wu Y-L, Zhang L, Fan Y et al. Randomized clinical trial of pembrolizumab vs chemotherapy for previously untreated Chinese patients with PD-L1-positive locally advanced or metastatic non-small-cell lung cancer: KEYNOTE-042 China Study. Int J Cancer 2021; 148: 2313-2320. doi: 10.1002/ijc.33399.

48: Yang J-J, Zhou Q, Liao R-Q et al. Nedaplatin/Gemcitabine Versus Carboplatin/Gemcitabine in Treatment of Advanced Non-small Cell Lung Cancer: A Randomized Clinical Trial. Chin J Cancer Res 2012; 24: 97-102. doi: 10.1007/s11670-012-0097-8.
